# Comorbidity and drug resistance of smear-positive pulmonary tuberculosis patients in the yi autonomous prefecture of China: a cross-sectional study

**DOI:** 10.1186/s12879-023-08568-3

**Published:** 2023-09-06

**Authors:** Tao Wang, Chaoxin Zhou, Lan Shang, Xiyuan Zhou

**Affiliations:** 1 Institute of Dermatology and Venereology, Sichuan Provincial People’s Hospital, University of Electronic Science and Technology, Chengdu, China; 2 Department of Radiology, The First People’s Hospital of Liangshan Yi Autonomous Prefecture, Xichang, Sichuan China; 3 Department of Radiology, Sichuan Provincial People’s Hospital, University of Electronic Science and Technology, Chengdu, China; 4 Chinese Academy of Sciences Sichuan Translational Medicine Research Hospital, Chengdu, 610072 China

**Keywords:** Sputum smear positive, Pulmonary tuberculosis, Impoverished area, Comorbidity, Drug-resistant

## Abstract

**Background:**

Tuberculosis (TB) has a high morbidity and mortality rate, and its prevention and treatment focus is on impoverished areas. The Liangshan Yi Autonomous Prefecture is a typical impoverished area in western China with insufficient medical resources and high HIV positivity. However, there have been few reports of TB and drug resistance in this area.

**Methods:**

We collected the demographic and clinical data of inpatients with sputum smear positive TB between 2015 and 2021 in an infectious disease hospital in the Liangshan Yi Autonomous Prefecture. Descriptive analyses were used for the epidemiological data. The chi-square test was used to compare categorical variables between the drug-resistant and drug-susceptible groups, and binary logistic regression was used to analyse meaningful variables.

**Results:**

We included 2263 patients, 79.9% of whom were Yi patients. The proportions of HIV (14.4%) and smoking (37.3%) were higher than previously reported. The incidence of extrapulmonary TB (28.5%) was high, and the infection site was different from that reported previously. When drug resistance gene detection was introduced, the proportion of drug-resistant patients became 10.9%. Patients aged 15–44 years (OR 1.817; 95% CI 1.162–2.840; P < 0.01) and 45–59 years (OR 2.175; 95% CI 1.335–3.543; P < 0.01) had significantly higher incidences of drug resistance than children and the elderly. Patients with a cough of ≥ 2 weeks had a significantly higher chance of drug resistance than those with < 2 weeks or no cough symptoms (OR 2.069; 95% CI 1.234–3.469; P < 0.01). Alcoholism (OR 1.741; 95% CI 1.107–2.736; P < 0.05) and high bacterial counts on sputum acid-fast smears (OR 1.846; 95% CI 1.115–3.058; P < 0.05) were significant in the univariate analysis.

**Conclusions:**

Sputum smear-positive TB predominated in Yi men (15–44 years) with high smoking, alcoholism, and HIV rates. Extrapulmonary TB, especially abdominal TB, prevailed. Recent drug resistance testing revealed higher rates in 15–59 age group and ≥ 2 weeks cough duration. Alcohol abuse and high sputum AFB counts correlated with drug resistance. Strengthen screening and supervision to curb TB transmission and drug-resistant cases in the region.

## Background

Tuberculosis (TB) is a major communicable disease worldwide, with high morbidity and mortality rates [1, 2]. According to a global report, 5.8 million patients were newly diagnosed in 2020 [3]. Before the COVID-19 pandemic, TB was the leading cause of death from an infectious disease [3, 4]. TB is not only a health problem driven by poverty; it can also exacerbate poverty. Moreover, TB is the key point affecting the Sustainable Development Goals of poverty eradication [5, 6]. Although high-income countries also have new TB cases annually, the World Health Organization (WHO)-listed high-burden countries are responsible for 80% of TB cases [3, 7]. Most of these countries have low or middle income [2]. Therefore, it is of great significance to control TB in poor areas. The WHO and the Chinese Center for Disease Control and Prevention have proposed long-term goals for TB prevention by 2030^3, 5^. However, as the top three high-burden countries in terms of TB, achieving the goal of ending the TB epidemic is challenging [8], especially in some impoverished areas in China where there are gatherings, underdeveloped healthcare resources, and high HIV prevalence.

The Liangshan Yi Autonomous Prefecture is located in southwest Sichuan Province and is the largest Yi community. The Yi people account for approximately 50% of the local permanent residents [9, 10]. It is the largest gathering place of the Yi minority in China. The Yi nationality is the sixth largest ethnic group in China and an ancient ethnic group [9]. The Yi people are accustomed to living in high-altitude areas and have their own language and eating habits [11]. Liangshan is one of the poorest areas in China, with poor local public health conditions [12]. By 2021, 11 of 17 counties were in severe poverty [13]. Therefore, it is among the regions with the highest HIV prevalence in China [14]. HIV infection affects all aspects of TB, including the development of active, extrapulmonary TB, increased mortality, and TB drug resistance [15–18]. A survival analysis of AIDS in this region showed that patients with TB had an increased mortality risk in recent years [19]. Despite being an area with a high incidence of infectious diseases, TB has rarely been reported.

We analysed the data of hospitalised patients to understand the epidemiological characteristics of sputum smear positive TB in this region. This type of TB is challenging to prevent and control as patients with sputum smear positive TB can expel *Mycobacterium tuberculosis* (*M. tuberculosis*) into the air by coughing, thus infecting others and spreading it further [20]. Especially in areas with high clusters and high HIV prevalence, the infection and transmission of TB are prevalent [3]. In addition, drug resistance is a key point that hinders the prevention and treatment of TB [3]. The most common mono-drug resistance reported previously is isoniazid resistance [21]. However, the annually increasing occurrence of multidrug-resistant TB (MDR-TB), which is resistant to both rifampicin and isoniazid, can cause great harm [22]. Some factors associated with resistance include poor patient compliance, alcohol abuse, smoking, co-infection, diabetes, HIV/AIDS, and acid-fast bacilli (AFB) smear positivity [23–25]. Therefore, this study summarised the occurrence of drug resistance and used logistic regression to analyse its risk factors in this group of patients.

## Methods

### Study area and population

We collected demographic and clinical data of patients with sputum smear positive TB who were hospitalised in the largest infectious disease hospital in the Liangshan Yi Autonomous Prefecture. The hospital is the only infectious disease hospital that provides tertiary care for patients with TB in the Liangshan Yi Autonomous Prefecture and is responsible for managing patients with TB in Liangshan city and surrounding regions. These clinical data came from the hospital ‘s his system, and included sex, age, ethnicity, disease course, living habits, comorbidities, smear results, and therapy. Living habits of alcohol consumption are defined here as chronic alcohol consumption and an average daily consumption of ≥ 15 g [26]. The resident population in this area is Yi and Han, with a ratio of approximately 1:1.

### Study design

#### Criteria for admission

**Fig. 1:**
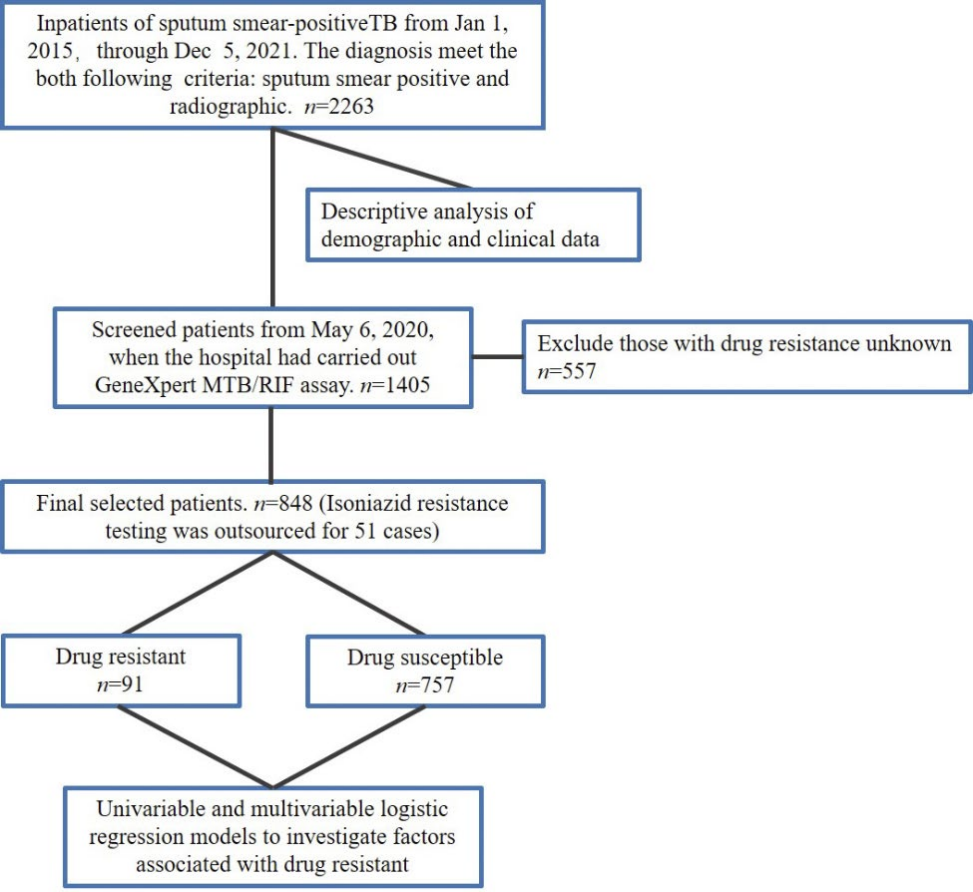
The process of data inclusion and exclusion

Data were collected from 1 to 2015 to 5 December 2021. Pulmonary TB and extrapulmonary TB diagnoses were made according to the Health Industry Standard of the People’s Republic of China - Diagnosis for pulmonary tuberculosis (WS 288–2017) and Classification of Tuberculosis (WS 196–2017). The diagnosis met the following two criteria: AFB smear-positive sputum and lung computed tomography supported the presence of active pulmonary TB [27]. Exclusion criteria included patients diagnosed with autoimmune diseases or a history of immunotherapy, those suffering from severe systemic diseases, and those with malignant tumors. HIV positivity was determined based on the initial test results upon admission, and individuals with a prior diagnosis or treatment for HIV were also excluded. Patients were categorized according to their Body Mass Index (BMI): those with a BMI less than 18.5 were considered ‘Underweight’, those with a BMI ranging from 18.5 to 24.9 were deemed ‘Normal weight’, while those with a BMI of 25 or more were classified as ‘Overweight’ [28]. At least two AFB sputum smear morning samples were collected from patients for testing. The Ziehl-Neelsen staining method and the grading standards promulgated by the National Health Commission of China were used for grading: Scanty, 1–8 on smear (300 fields examined); +, 3–9 in 100 fields (300 fields examined); 2+, 1–9 in 10 fields (100 fields examined); 3+, 1–9 per field of vision (50 fields examined); and 4+, ≥ 10 per field of vision (50 fields examined). For the analysis, we used the age groups 0–14, 15–44, 45–59, and > 60 years. Patients were classified into three groups according to whether they had a cough persisting for more than two weeks. As per WHO guidelines, such patients warrant further investigation and screening in regions with high TB prevalence [29]. Unrecorded values are represented as missing values. Following the hospital’s execution of genetic testing for drug-resistant mutations on May 6, 2020, we proceeded with patient screening. Under constrained conditions, the GeneXpert MTB/RIF assay used in hospitals facilitated the detection of Rifampicin resistance across all patients [2, 30]. Additionally, for the purpose of Isoniazid resistance genetic testing, samples from a subset of patients were dispatched. The drug resistance screening took place before the initiation of treatment. First-line treatment was the preferred option upon tuberculosis diagnosis. In cases of recurrent tuberculosis or rifampin-resistant and multi-drug resistant tuberculosis, second-line drug treatment was selected. After further excluding patients with unknown data, they were assigned to the drug-resistant and drug-susceptible groups according to the drug-susceptibility test results. The inclusion and exclusion data are illustrated in Fig. 1.

### Statistical methods

Descriptive analyses were performed on the collected clinical data of all hospitalised patients, consistent with sputum smear positive TB. Age was not considered a continuous variable, but the patients were divided into different age groups. The chi-square test was used to compare categorical variables between the drug-resistant and drug-susceptible groups. Statistical significance was set at *P* < 0.05, but the binary logistic regression model included variables with *P <* 0.1. To identify significant variables affecting drug resistance, we used both univariate and multivariate binary logistic regression (backward stepwise logistic regression) to compare the included variables. A dummy variable for age-grouped was an ‘indicator-first’ of contrast selection, while cough selection was ‘deviation-first’. The TB smear was classified, and only 4 + positive patients were counted to determine whether the quantity of multiple bacteria affected drug resistance. Statistical analyses were performed using SPSS version 23.0 and GraphPad Prism 7.04 (GraphPad Software Inc., CA, USA).

### Ethics statement

The study collected data from the patient’s hospital records and anonymised them. The acquisition of all clinical data and laboratory results, as well as the waiver of patient informed consent has been approved by the Ethics Review Committee of Sichuan Provincial Academy of Medical Sciences and Sichuan Provincial People’s Hospital (Protocol 20220-254).

## Results

### Characteristics of patients with sputum smear positive TB

**Fig. 2:**
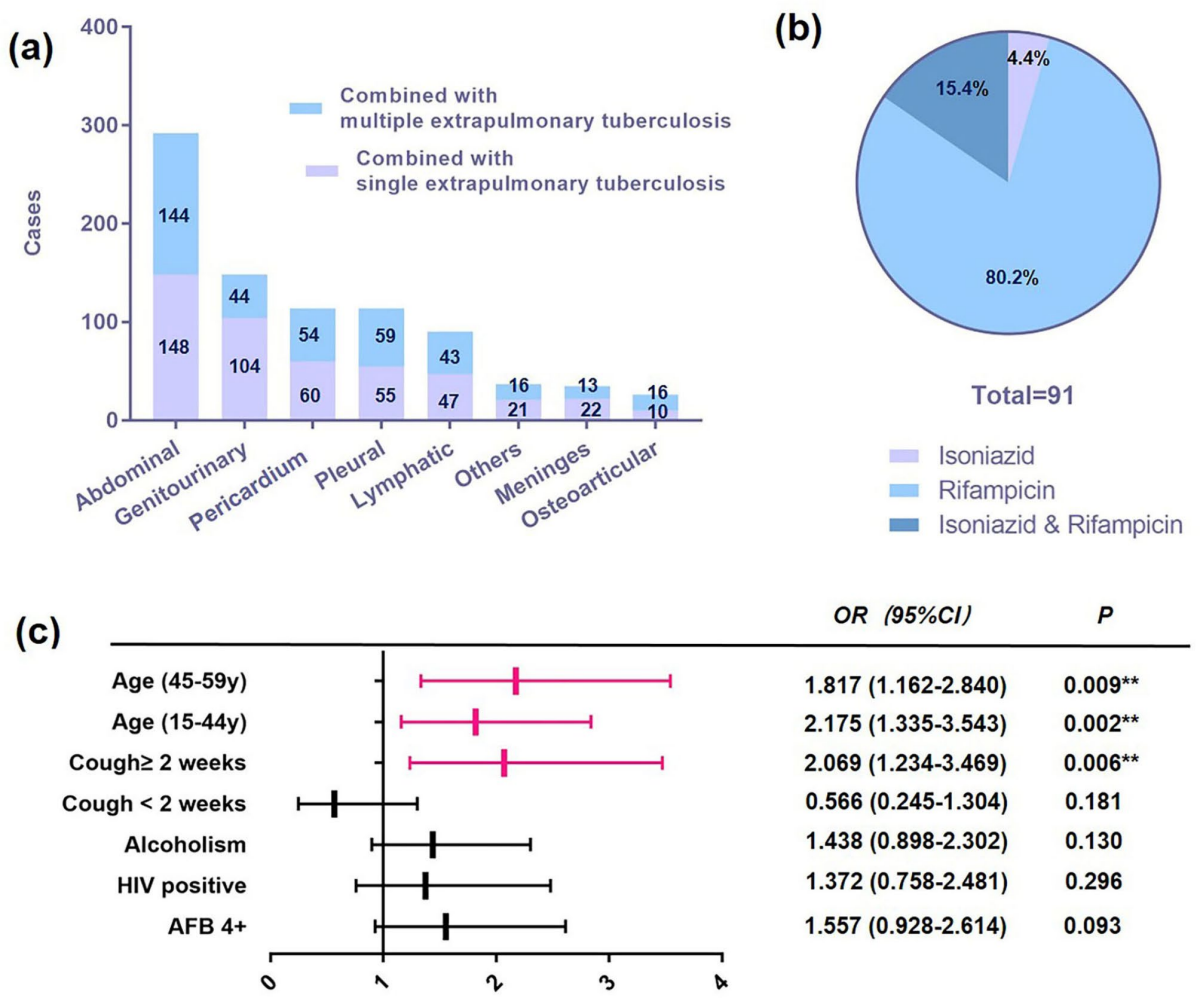
Extrapulmonary TB-affected sites, specific drug resistance ratios, and forest map of drug resistance-related risk factors. **(a)** The top five most common extrapulmonary tuberculosis (TB) were abdominal, genitourinary, pericardium, pleural, and lymphatic. **(b)** A total of 91 drug-resistant patients, 73 (80.2%) were resistant to rifampicin, 4 (4.4%) were resistant to isoniazid, and 14 (15.4%) were resistant to both. **(c)** The 15–59-year-old group developed drug resistance about two times as high as children and the elderly. A cough duration of ≥ 2 weeks was also a risk factor for drug resistance (OR 1.999; 95% CI 1.189–3.361; *P* < 0.01)

During the data collection period, 2263 patients with sputum smear positive TB were hospitalised (Table 1). Among these patients, 63.2% were male, and the majority belonged to the Yi ethnic group (79.9%). Age distribution ranged from 1 month to 94 years, with the highest proportion (59.3%) being in the 15–44 age group, followed by 45–59-year-olds (19.1%), the elderly (17.9%), and children aged 0–14 years (4.1%). In terms of body weight status, 41.9% of patients were classified as underweight, 53.2% as normal weight, and 4.9% as overweight or obese. A history of smoking was observed among 37.3% of patients, while 26.7% had a history of alcoholism. Patients with type 2 diabetes accounted for 8.2% and HIV-positive patients accounted for 14.4%. Patients with extrapulmonary TB accounted for 28.5%, of whom 26.2% had multiple sites. The top five most common extrapulmonary TB cases were abdominal, genitourinary, pericardial, pleural, and lymphatic (Fig. 2A). Regarding treatment, 84.3% of the patients were administered first-line anti-TB therapy, whereas 7.1% were administered second-line anti-TB therapy. Another 8.7% of the patients had poor adherence, refusal, or discontinuation of the medication.

**Table 1:** Characteristics of 2263 tuberculosis patients in a infectious disease hospital of Liangshan Yi Autonomous Prefecture

Characteristic	Value (n = 2263)
**Gender-n. (%)**	
** Male**	1430 (63.2)
** Female**	833 (36.8)
**Age-Years**	
** <14-n. (%)**	92 (4.1)
** 15-44-n. (%)**	1342 (59.3)
** 45-59-n. (%)**	433 (19.1)
** ≥ 60-n. (%)**	405 (17.9)
**Ethnicity-n. (%)**	
** Han**	455 (20.1)
** Yi**	1808 (79.9)
**BMI**	
** < 18.5-n. (%)**	948 (41.9)
** 18.5-24.9-n. (%)**	1203 (53.2)
** ≥ 25-n. (%)**	112 (4.9)
**Alcoholism -n. (%)**	
** Yes**	604 (26.7)
** No**	1644 (72.6)
** Missing**	15 (0.7)
**Comobidites-n. (%)**	
** COPD**	142 (6.3)
** Type 2 diabetes**	185 (8.2)
** HIV positive**	326 (14.4)
** Extrapulmonary tuberculosis**	644 (28.5)
**Treatment**	
** First-line anti-TB drugs**	1903 (84.3)
** Second-line anti-TB drugs**	176 (7.8)
** Non-compliance with medical advice**	184 (8.1)
**Drug resistance**	
** Yes**	98 (4.3)
** Drug susceptible**	1438 (63.5)
** Not clear**	727 (32.1)

### Characteristics of drug-resistant and drug-susceptible groups

The GeneXpert MTB/RIF assay was introduced in this hospital in May 2020. Out of a total of 2263 patients, 1405 were selected for the screening process. Data loss accounted for 557 (39.6%) of these selected patients, resulting in 848 remaining patients for analysis. Out of these, 51 also underwent isoniazid resistance testing by a separate company. Upon completion of all tests, drug resistance was identified in 91 patients, leading to an incidence rate of 10.7%. Further breakdown of these drug-resistant cases revealed that 73 (80.2%) were resistant to rifampicin, 4 (4.4%) to isoniazid, and 14 (15.4%) demonstrated resistance to both drugs, thus categorized as multi-drug resistant tuberculosis (MDR-TB) (Fig. 2B). Finally, 91 and 747 patients were included in the drug-resistant and drug-susceptible groups, respectively. There were no significant differences in sex, ethnicity, smoking, HIV positivity, extrapulmonary TB, or sputum smear between the two groups (Table 2). However, the *P*-value for HIV-positive and sputum smear results was < 0.1. There were statistically significant differences in age stratification (*P* < 0.001), alcohol consumption (*P* < 0.05), and cough status (*P* < 0.001) (Table 2).

**Table 2:** Demograhic and clinical characteristics after the hospital carried out drug resistance gene detection in 2020

Variable	Patients, No.(%)		
	Drug-resistant (*n* = 91)	Drug-susceptible (*n* = 757)	*P*-value
**Age-years**			0.000***
≤ **14**	**1 (1.1)**	**38 (5.0)**	
** 15–44**	**63 (69.2)**	**449 (59.2)**	
** 45–59**	**25 (27.5)**	**136 (18.0)**	
** ≥ 60**	**2 (2.2)**	**135 (17.8)**	
Male	62 (68.1)	464 (61.3)	0.204
Yi Ethnicity	65 (71.4)	554 (73.2)	0.722
Smoke	38 (41.8)	283 (37.4)	0.417
**Alcoholism**	**35 (38.5)**	**200 (26.4)**	0.015*
**HIV positive**	**17 (18.7)**	**89 (11.8)**	**0.059#**
Compicated with extrapulmonary TB	14 (15.4)	169 (22.3)	0.128
**Cough**			0.000***
** No**	**5 (5.5)**	**100 (13.2)**	
** Cough for<2 weeks**	**3 (3.3)**	**81 (10.7)**	
** Cough for ≥ 2 weeks**	**83 (91.2)**	**576 (76.1)**	
**AFB sputum smear**			**0.099#**
** Scanty**	**5 (5.5)**	**58 (7.7)**	
** +**	**23 (25.3)**	**264 (34.9)**	
** 2+**	**22 (24.2)**	**188 (24.8)**	
** 3+**	**17 (18.7)**	**124 (16.4)**	
** 4+**	**24 (26.4)**	**123 (16.2)**	

TB: tuberculosis; AFB: acid-fast bacilli

*: *P*-value< 0.05; **: *P*-value< 0.01; ***: *P*-value< 0.001; #: *P*-value< 0.1

### Risk factor characteristics of drug-resistant patients with TB

After including significant variables in the binary regression model, we summarised the risk of developing resistance for these variables (Table 3; Fig. 2C). Young patients aged 15–44 (multivariate analysis: OR 1.817; 95% CI 1.162–2.840; *P* < 0.01) and middle-aged patients aged 45–59 (multivariate analysis: OR 2.175; 95% CI 1.335–3.543; *P* < 0.01) were more likely to develop drug resistance than children and the elderly. A cough duration of ≥ 2 weeks was a risk factor for drug resistance relative to no cough and cough for < 2 weeks (multivariate analysis: OR 2.069; 95% CI 1.234–3.469; *P* < 0.01). These risk factors were meaningful in univariate and multivariate logistic regression analyses. However, alcohol (univariate analysis: OR 1.741; 95% CI 1.107–2.736; *P* < 0.05) and high bacterial counts in sputum smears (univariate analysis: OR 1.846; 95% CI 1.115–3.058; *P* < 0.05) were the only risk factors in univariate logistic regression but were not risk factors in multivariate logistic regression.

**Table 3:** Univariate and Multivariate logistic regression assessing risk factors for drug resistance of tuberculosis

Variable	Univariate		Multivariate	
	OR (95% CI)	*P*-value	OR (95% CI)	*P*-value
**Age-years#**				
<14 & ≥60	Ref	0.001**	Ref	0.003**
** 15–44**	**8.045 (2.493–25.958)**		**1.817 (1.162–2.840)**	
** 45–59**	**10.539 (3.116–35.645)**		**2.175 (1.335–3.543)**	
**Alcoholism**	**1.741 (1.107–2.736)**	0.016*	1.438 (0.898–2.302)	0.130
HIV positive	1.724 (0.973–3.054)	0.062	1.372 (0.758–2.481)	0.296
**Cough##**				
No	Ref	0.008**	Ref	0.022*
Cough for<2 weeks	0.575 (0.251–1.317)		0.566 (0.245–1.304)	
Cough for ≥ 2 weeks	**2.238 (1.343–3.730)**		**2.069 (1.234–3.469)**	
**AFB smear**				
** 4+**	**1.846 (1.115–3.058)**	0.017*	1.557 (0.928–2.614)	0.093

AFB: acid-fast bacilli

*: *P*-value< 0.05; **: *P*-value< 0.01; ***: *P*-value< 0.001

#: contrast selection of dummy variables is “indicator-first”; ##: contrast selection of dummy variables is “deviation-

## Discussion

The 2263 patients with sputum smear positive TB were mainly Yi men aged 15–44 years, and sex characteristics were consistent with the previous reported male-to-female ratio of 2:1 [1, 4, 31]. However, compared with other regions of China or other ethnic minorities with tuberculosis, these patients are generally younger [32–35]. In contrast to the predominant pattern of China’s pulmonary TB group, which primarily affects individuals over 65 years of age [32], our study reveals a significant proportion (59.3%) of patients aged 15–44. This discrepancy may be attributed to increased social activity within this age group, potentially exposing them to risk factors such as smoking, alcohol consumption, and HIV infection. Furthermore, our findings indicate differences in body mass index (BMI) compared to previous reports [36]. Notably, we observed a relatively large proportion of underweight patients, possibly linked to the limited access to medical resources in the region. It is noteworthy that hospitalized patients in this area commonly exhibit evident and severe symptoms. In our survey, we also observed that the number of smokers, alcoholism, and HIV-positive people are higher than those in previous surveys [31, 37–39]. All these inconsistencies can be explained by approximately 80% of the Yi patients in the study group. This ratio far exceeds the 48.9% of the local Yi nationality resident population [10]. Unlike other nomadic minorities, the Yi people live in remote villages in the high-altitude mountains with their families [11]. These mountain villages have poor sanitary conditions [12, 40]. According to previous studies, the incidence of tuberculosis in Liangshan has shown a clustering trend, mainly concentrated in areas dominated by Yi ethnic groups [41]. This results in the majority of hospitalized patients being Yi people. They mainly eat potatoes, oats, and buckwheat, eat little meat, and like to smoke and drink alcohol [42]. Smoking and alcohol consumption are risk factors that cause great harm if they occur simultaneously [43, 44]. The most important risk factor for TB is HIV positivity, which is also high in this nation. The incidence of HIV and TB co-infection in our study was 14.14%, which is higher than the global rate of 9% and China’s rate of 7.4% [39, 45].

Because of these risk factors, as well as the remoteness of living, these patients visit the hospital only after the symptoms manifest and significantly affect their work. In our study, more than 1/4 of the patients with extrapulmonary TB had complications at more than two sites, which was also higher than 14% in other data reported in China [46]. Moreover, the characteristics of this group of patients with extrapulmonary TB are very different from those previously reported. Firstly, the incidence of extrapulmonary TB is 28.5%, which is significantly higher than the values previously reported globally (14%) and in China (8.5%) [27, 47]. Secondly, the occurrence sites of extrapulmonary TB identified in this study differed from those commonly reported previously, such as the lymph nodes and skeletal system [27, 48]. The most common sites in our patients were in the abdomen (34.1%) and genitourinary system (17.3%), which is also inconsistent with the most common extrapulmonary TB in China in the pleura (49.8%) and bronchial TB (14.8%) [46]. Compared with genitourinary TB, which is also a common extrapulmonary TB [49], the incidence of abdominal TB is generally 1–3% [50]. The reported rates of extrapulmonary TB globally and in China were 12% and 4.8%, respectively [46, 51]. Gastrointestinal TB can spread through direct ingestion or distant spread in the blood, lymph, or adjacent tissues. Direct ingestion can be through sputum or contaminated food [52]. The Yi people exhibit communal dining practices, where multiple individuals share a single bowl to consume both food and alcohol. This tradition disregards the use of individual tableware and often involves eating with hands, contributing to suboptimal hygiene practices. These may be the reasons for the high rate of abdominal TB, consistent with the high incidence in developing countries of low socioeconomic status [53]. In addition, HIV positivity is also a health factor for abdominal TB [54].

In this study, 15.9% of the patients were on second-line anti-TB treatment or had received no treatment because of poor compliance. Although the drugs for anti-TB treatment are free, travel expenses, examination fees, and lost work expenses remain a heavy burden for patients. Patients generally choose to stop medication after the symptoms have improved slightly, thus resulting in drug resistance and further aggravating the difficulty of treatment [55]. Approximately 10.7% of the patients in our study were drug-resistant, which is lower than the current global and domestic levels of drug-resistant TB [56]. However, this does not reflect the drug resistance of TB in this region because testing for drug resistance has been lacking. The hospital did not use rapid genetic detection of TB drug resistance until 2020, while the technology has been in use for almost 20 years [57]. The traditional drug-susceptibility test for TB is not applicable in this region because of the long wait for culture results. Previous records of drug resistance and patients receiving second-line therapy have relied on physicians’ clinical experience. Among 91 cases of detected drug resistance, 80.2% were rifampicin-resistant (RR) TB and 15.4% were MDR-TB. This is different from isoniazid resistance predominant, and 80% of the RR eventually become MDR [25, 58]. This difference may be due to a lack of laboratory tests.

In our regression model for the analysis of drug resistance-related risk factors, we still obtained the impact of living habits and poverty. The entire group of patients who are family labourers have a wide range of social activities and a significant increase in the incidence of drug resistance. Patients aged 15–44 and 45–59 years were 1.817 and 2.175 times more likely to develop drug resistance than children and the elderly, respectively. In studies of TB drug resistance in other regions of China, the highest incidence was found for those with low education levels, with low income, and aged 40–65 [59]. Because this age group is the source of the labour force at home, they may be forced to give up treatment for reasons such as continuing work and economic reasons, and compliance is poor. Although HIV is closely related to drug resistance in previous reports [17] and is most frequently seen in the age group of 15–44 years [14], our study did not identify it as a risk factor for TB drug resistance. Previous studies have shown that drug resistance mainly occurs in low-education, low-income, and poor populations [25]. Our multivariable analysis revealed that patients who had a cough for ≥ 2 weeks were 2.08 times more likely to develop drug resistance than those without a cough and within 2 weeks of duration of illness. This finding reaffirms the validity of the erstwhile WHO guideline, which identified a persistent cough exceeding two weeks as a typical symptoms of tuberculosis [60]. Despite subsequent amendments to this criterion, it continues to serve as a significant marker, particularly in regions exhibiting high prevalence rates [29]. The results of our study emphasize the relevance of this 2-week threshold in identifying potential TB cases early and possibly reducing the likelihood of developing drug-resistant forms of the disease. These two risk factors for TB drug resistance explain the significant impact of the backward economy, poverty, and inconvenience of medical treatment in this region. The other two risk factors that only appeared in the univariate regression analysis were alcohol consumption and high bacterial count in the AFB smear test. Combined alcohol abuse, low education, and low income are predictors of MDR [61]. Moreover, a high bacterial count in the AFB smear test might indicate a high organism burden and, thus, greater susceptibility to resistance [62, 63]. However, it is also believed that a large number of acid-fast bacilli results from drug resistance rather than the cause [64]. Therefore, it is essential to grade the initial sputum smear before treatment [63].

Our study only investigated hospitalised patients in an infectious disease hospital in the Liangshan Yi Autonomous Prefecture. Owing to poverty, poor sanitary conditions, and inconvenient medical treatment in the local area, many patients may seek medical treatment only when the disease is serious. This can lead to bias in patient selection and the absence of many patients with sputum smear positive TB. In addition, the drug resistance testing conducted in this hospital was not comprehensive and may be considered less advanced, which could potentially impact the accuracy of the regression model results. The deficiency in comprehensiveness was further magnified due to our reliance on an external company for the Rifampicin resistance test. This test was only performed on a limited sample of 51 patients, thereby intensifying the perceived inadequacy. However, this hospital accommodates most local inpatients with TB, and the epidemiological characteristics obtained and risk factors for TB drug resistance were within the interpretable range. Hopefully, these data will help to understand the clinical and treatment status of locally sputum smear positive TB and further help local TB prevention and treatment. These data will contribute to a better understanding of the clinical and treatment status of locally sputum smear-positive TB, facilitating targeted approaches for TB prevention and treatment. For instance, identifying age groups with high drug resistance incidence calls for intensified drug resistance screening and long-term medication follow-up. Furthermore, addressing the high incidence of abdominal extrapulmonary tuberculosis and TB clustering requires regular patient education and health diet guidance.

## Conclusions

Patients with sputum smear positive TB who were hospitalised at the Liangshan Yi Autonomous Prefecture infectious disease hospital were mainly Yi men aged 15–44 years. Smoking, alcoholism, and HIV positivity had the highest proportions. The incidence of extrapulmonary TB is higher than in other regions in China, and the incidence site is different; abdominal TB is the most common. Because of local poverty and poor medical conditions, drug resistance gene testing has been recently carried out. The 15–59-year-old age group developed drug resistance two times higher than children and the elderly. A cough duration of ≥ 2 weeks was also a risk factor for drug resistance. In addition, alcohol abuse and high bacterial counts in sputum AFB smears were also associated with drug resistance. Based on these characteristics, local governments, disease control authorities, and hospitals can enhance screening and supervision measures. The ultimate goal is to mitigate the rate of tuberculosis transmission and reduce the occurrence of drug-resistant tuberculosis within the region.

## Data Availability

The datasets generated and/or analysed during the current study are not publicly available, but are available from the corresponding author on reasonable request.

## Declarations

### Statements

All methods were carried out in accordance with relevant guidelines and regulations.

## List of abbreviations

TB: Tuberculosis
HIV: Human Immunodeficiency Virus
OR: Odds Ratio
CI: Confidence Interval
AIDS: Acquired Immure Deficiency Syndrome
MDR-TB: multidrug-resistant TB
AFB: Acid-fast Bacilli
RR: Rifampicin-resistant

## References

[1] Pai M, Behr MA, Dowdy D, Dheda K, Divangahi M, Boehme CC (2016). Tuberculosis Nat Rev Dis Primers.

[2] Furin J, Cox H, Pai M, Tuberculosis (2019). The Lancet.

[3] Global Tuberculosis Report. 2021. World Health Organization; Geneva: World Health Organizationaccessed 21 January 2022.

[4] Floyd K, Glaziou P, Zumla A, Raviglione M (2018). The global tuberculosis epidemic and progress in care, prevention, and research: an overview in year 3 of the end TB era. The Lancet Respiratory Medicine.

[5] Long Q, Guo L, Jiang W, Huan S, Tang S (2021). Ending tuberculosis in China: health system challenges. The Lancet Public Health.

[6] Rai B, Dixit K, Aryal TP, Mishra G, Siqueira-Filha NT, Paudel PR et al. Developing feasible, locally appropriate socioeconomic support for TB-Affected Households in Nepal. Trop Med Infect Dis. 2020;5(2). 10.3390/tropicalmed5020098 PMC7345977 32532101

[7] Global Tuberculosis Report. 2020. Geneva: World Health Organization.: Published 2020. Accessed October 20, 2020. ; 2020.

[8] Long Q, Jiang WX, Zhang H, Cheng J, Tang SL, Wang WB (2021). Multi-source financing for tuberculosis treatment in China: key issues and challenges. Infect Dis Poverty.

[9] Cheng J, Song B, Fu J, Zheng X, He T, Fu J (2021). Genetic polymorphism of 19 autosomal STR loci in the Yi ethnic minority of Liangshan Yi autonomous prefecture from Sichuan province in China. Sci Rep.

[10] Shan G, Wei D, Wang C, Zhang J, Wang B, Ma M (2011). Trends of overweight and obesity in Yi people between 1996 and 2007: an Yi migrant study. Biomed Environ Sci.

[11] Shan G, Wei D, Wang C, Zhang J, Wang B, Ma M (2012). Body Mass Index and Hypertension hemodynamic subtypes in Yi Farmers and Migrants. Biomed Environ Sci.

[12] Liao RJ, Ji-Ke CN, Zhang T, Liao Q, Li L, Zhu TY (2020). Coronavirus disease 2019 epidemic in impoverished area: Liangshan Yi autonomous prefecture as an example. Infect Dis Poverty.

[13] Liao R, Hu L, Liao Q, Zhu T, Yang H, Zhang T (2022). Analysis of death causes of residents in poverty-stricken Areas in 2020: take Liangshan Yi Autonomous Prefecture in China as an example. BMC Public Health.

[14] Yang H, Xie X, Nie A, Yin Y, Wang H, Chen H (2020). HIV-Related Stigma among People living with HIV in Liangshan Yi Autonomous Prefecture, China. J Assoc Nurses AIDS Care.

[15] Naing C, Mak JW, Maung M, Wong SF, Kassim AI (2013). Meta-analysis: the association between HIV infection and extrapulmonary tuberculosis. Lung.

[16] Mendez-Samperio P (2017). Diagnosis of tuberculosis in HIV co-infected individuals: current status, Challenges and Opportunities for the future. Scand J Immunol.

[17] Khan PY, Yates TA, Osman M, Warren RM, van der Heijden Y, Padayatchi N (2019). Transmission of drug-resistant tuberculosis in HIV-endemic settings. Lancet Infect Dis.

[18] Scully EP, Bryson BD. Unlocking the complexity of HIV and Mycobacterium tuberculosis coinfection. J Clin Invest. 2021;131(22). 10.1172/JCI154407 PMC8592542 34779416

[19] Zhang GGY, Wang Q, Zhang S, Liao Q, Yu G, Wang K, Wang J, Ye S, Liu Z (2015). Survival time and associated factors of 8 310 AIDS patients initially receiving antiretroviral treatment of Liangshan Yi Autonomous Prefecture, Sichuan province of China. Zhonghua Yu Fang Yi Xue Za Zhi.

[20] Brett KD, Severn C. M. Identification of Tuberculosis: A Review of the Guidelines [Internet]. Ottawa (ON): Canadian Agency for Drugs and Technologies in Health. 2020;2020. 33074601

[21] Chien JY, Chen YT, Wu SG, Lee JJ, Wang JY, Yu CJ (2015). Treatment outcome of patients with isoniazid mono-resistant tuberculosis. Clin Microbiol Infect.

[22] Lange C, Chesov D, Heyckendorf J, Leung CC, Udwadia Z, Dheda K (2018). Drug-resistant tuberculosis: an update on disease burden, diagnosis and treatment. Respirology.

[23] Nguyen L (2016). Antibiotic resistance mechanisms in M. tuberculosis: an update. Arch Toxicol.

[24] Hameed HMA, Islam MM, Chhotaray C, Wang C, Liu Y, Tan Y (2018). Molecular targets related Drug Resistance Mechanisms in MDR-, XDR-, and TDR-Mycobacterium tuberculosis strains. Front Cell Infect Microbiol.

[25] Singh R, Dwivedi SP, Gaharwar US, Meena R, Rajamani P, Prasad T (2020). Recent updates on drug resistance in Mycobacterium tuberculosis. J Appl Microbiol.

[26] Chinese Society of Nutrition: Dietary Guidelines for Chinese residents. (2022), 1 edition. Bei Jing: People’s Medical Publishing House; 2022.

[27] Pang Y, An J, Shu W, Huo F, Chu N, Gao M (2019). Epidemiology of Extrapulmonary Tuberculosis among Inpatients, China, 2008–2017. Emerg Infect Dis.

[28] Physical status (1995). The use of and interpretation of anthropometry. Report of a WHO expert committee. World Health Organ Tech Rep Ser.

[29] World Health Organization (2016) Systematic screening for active tuberculosis: principles and recommendations. http://www.who.int/tb/tbscreening/en/. Accessed August 18, 2016. 25996015

[30] Nachega JBC (2003). Tuberculosis drug resistance: A global threat. Clin Infect Dis.

[31] Winter JR, Smith CJ, Davidson JA, Lalor MK, Delpech V, Abubakar I (2020). The impact of HIV infection on tuberculosis transmission in a country with low tuberculosis incidence: a national retrospective study using molecular epidemiology. BMC Med.

[32] Kang W, Du J, Yang S, Yu J, Chen H, Liu J (2021). The prevalence and risks of major comorbidities among inpatients with pulmonary tuberculosis in China from a gender and age perspective: a large-scale multicenter observational study. Eur J Clin Microbiol Infect Dis.

[33] Gilmour B, Xu Z, Bai L, Alene KA, Clements ACA (2022). The impact of ethnic minority status on tuberculosis diagnosis and treatment delays in Hunan Province, China. BMC Infect Dis.

[34] Xu J, Tang W, Cheng S, Mahapatra T, Zhou L, Lai Y (2014). Prevalence and predictors of HIV among chinese tuberculosis patients by provider-initiated HIV testing and counselling (PITC): a multisite study in South Central of China. PLoS ONE.

[35] Li T, Cheng Q, Li C, Stokes E, Collender P, Ohringer A (2019). Evidence for heterogeneity in China’s progress against pulmonary tuberculosis: uneven reductions in a major center of ongoing transmission, 2005–2017. BMC Infect Dis.

[36] Badawi A, Gregg B, Vasileva D (2020). Systematic analysis for the relationship between obesity and tuberculosis. Public Health.

[37] Zhang H, Xin H, Li X, Li H, Li M, Lu W (2017). A dose-response relationship of smoking with tuberculosis infection: a cross-sectional study among 21008 rural residents in China. PLoS ONE.

[38] Chin DP, Crane CM, Diul MY, Sun SJ, Agraz R, Taylor S (2000). Spread of Mycobacterium tuberculosis in a community implementing recommended elements of Tuberculosis Control. JAMA.

[39] Meintjes G, Brust JCM, Nuttall J, Maartens G (2019). Management of active tuberculosis in adults with HIV. The Lancet HIV.

[40] Li TYC, He JG, Li YK, Xiao Y, Li J, Wang DX, Chen C, Wu JL (2017). Spatial-temporal distribution of smear positive pulmonary tuberculosis in Liangshan Yi autonomous prefecture, Sichuan province, 2011–2016. Zhonghua Liu Xing Bing Xue Za Zhi.

[41] Li T, Yang CH, He JG, Li YK, Xiao Y, Li J (2017). Spatial-temporal distribution of smear positive pulmonary tuberculosis in Liangshan Yi autonomous prefecture, Sichuan province, 2011–2016. Zhonghua Liu Xing Bing Xue Za Zhi.

[42] Long C, Li S, Long B, Shi Y, Liu B (2009). Medicinal plants used by the Yi ethnic group: a case study in central Yunnan. J Ethnobiol Ethnomed.

[43] Silva DR, Munoz-Torrico M, Duarte R, Galvao T, Bonini EH, Arbex FF (2018). Risk factors for tuberculosis: diabetes, smoking, alcohol use, and the use of other drugs. J Bras Pneumol.

[44] Hermosilla S, You P, Aifah A, Abildayev T, Akilzhanova A, Kozhamkulov U (2017). Identifying risk factors associated with smear positivity of pulmonary tuberculosis in Kazakhstan. PLoS ONE.

[45] Gao L, Zhou F, Li X, Jin Q (2010). HIV/TB co-infection in mainland China: a meta-analysis. PLoS ONE.

[46] Kang W, Liu S, Du J, Tang P, Chen H, Liu J (2022). Epidemiology of concurrent extrapulmonary tuberculosis in inpatients with extrapulmonary tuberculosis lesions in China: a large-scale observational multi-centre investigation. Int J Infect Dis.

[47] Rodriguez-Takeuchi SY, Renjifo ME, Medina FJ (2019). Extrapulmonary Tuberculosis: pathophysiology and imaging findings. Radiographics.

[48] Leeds IL, Magee MJ, Kurbatova EV, del Rio C, Blumberg HM, Leonard MK (2012). Site of extrapulmonary tuberculosis is associated with HIV infection. Clin Infect Dis.

[49] Abbara A, Davidson RN (2011). Medscape. Etiology and management of genitourinary tuberculosis. Nat Rev Urol.

[50] Eraksoy H (2021). Gastrointestinal and abdominal tuberculosis. Gastroenterol Clin North Am.

[51] Wolde TG, Huang S, Zhang K, Wu J, Gao W, Li Q (2021). Evaluation of twenty-one cases of abdominal tuberculosis: a single-center experience. Surg Infect (Larchmt).

[52] Lee WK, Van Tonder F, Tartaglia CJ, Dagia C, Cazzato RL, Duddalwar VA (2012). CT appearances of abdominal tuberculosis. Clin Radiol.

[53] Udgirkar S, Jain S, Pawar S, Chandnani S, Contractor Q, Rathi P (2019). Clinical Profile, Drug Resistance Pattern and Treatment Outcomes of Abdominal Tuberculosis Patients in western India. Arq Gastroenterol.

[54] Almadi MA, Aljebreen AM, Sanai FM, Marcus V, Almeghaiseeb ES, Ghosh S (2011). New insights into gastrointestinal and hepatic granulomatous disorders. Nat Rev Gastroenterol Hepatol.

[55] Mirzayev F, Viney K, Linh NN, Gonzalez-Angulo L, Gegia M, Jaramillo E et al. World Health Organization recommendations on the treatment of drug-resistant tuberculosis, 2020 update. Eur Respir J. 2021;57(6). 10.1183/13993003.03300-2020 PMC8176349 33243847

[56] Long Q, Qu Y, Lucas H (2016). Drug-resistant tuberculosis control in China: progress and challenges. Infect Dis Poverty.

[57] Y M. Progress and application of molecular genetics in the research of Mycobacterium tuberculosis. Kekkaku. 1993;68(11):709–14. 8264127

[58] Kwak SH, Choi JS, Lee EH, Lee SH, Leem AY, Lee SH (2020). Characteristics and treatment outcomes of Isoniazid Mono-Resistant tuberculosis: a retrospective study. Yonsei Med J.

[59] Qi Z, Gao X, Wang YF, Liu C (2020). Epidemic characteristics and drug resistance of tuberculosis in North China. Heliyon.

[60] World Health Organization. Systematic screening for active tuberculosis: principles and recommendations. WHO Press; 2013. 25996015

[61] Di Gennaro F, Pizzol D, Cebola B, Stubbs B, Monno L, Saracino A (2017). Social determinants of therapy failure and multi drug resistance among people with tuberculosis: a review. Tuberculosis (Edinb).

[62] Conaty SJ, Hayward AC, Story A, Glynn JR, Drobniewski FA, Watson JM (2004). Explaining risk factors for drug-resistant tuberculosis in England and Wales: contribution of primary and secondary drug resistance. Epidemiol Infect.

[63] Horne DJ, Johnson CO, Oren E, Spitters C, Narita M (2010). How soon should patients with smear-positive tuberculosis be released from inpatient isolation?. Infect Control Hosp Epidemiol.

[64] Elmi OS, Hasan H, Abdullah S, Mat Jeab MZ, Bin Alwi Z, Naing NN (2015). Multidrug-resistant tuberculosis and risk factors associated with its development: a retrospective study. J Infect Dev Ctries.

